# Impact of Frequent ARID1A Mutations on Protein Stability: Insights into Cancer Pathogenesis

**DOI:** 10.21203/rs.3.rs-5225582/v1

**Published:** 2024-12-19

**Authors:** Rajen K Goutam, Gangtong Huang, Exequiel Medina, Feng Ding, William J. Edenfield, Hugo Sanabria

**Affiliations:** 1. Department of Physics and Astronomy, Clemson University, Clemson, 29634, SC, USA; 2. Departamento de Bioquímica y Biología Molecular, Facultad de Ciencias Químicas y Farmacéuticas, Universidad de Chile, Chile.; 3. Institute for Translational Oncology Research, Prisma Health, Greenville, SC, USA.

## Abstract

The ARID1A gene, frequently mutated in cancer, encodes the AT-rich interactive domain-containing protein 1A, a key component of the chromatin remodeling SWI/SNF complex. The ARID1A protein features a conserved DNA-binding domain (ARID domain) of approximately 100 residues crucial for its function. Despite the frequency of mutations, the impact on ARID1A’s stability and contribution to cancer progression remains unclear. We analyzed five frequent missense mutations R1020S, M1022K, K1047Q, G1063V, and A1089T identified in The Cancer Genome Atlas (TCGA) to assess their effects on the stability of the ARID domain using a hybrid experimental and computational approach. By combining computational stability from web server tools, the structural dynamics from replica exchange discrete molecular simulation (rexDMD), and thermal and chemical denaturation experiments, we found that the R1020S mutation severely decreases structural stability, making it the most impactful, while M1022K has minimal effect, and others lie in between. These findings enhance our understanding of the structural-functional relationship of ARID1A missense mutations at the molecular levels and their role in cancer pathogenesis. This research paves the way for identifying and categorizing which ARID1A mutations are most pathogenic, potentially guiding the development of targeted therapies tailored to specific mutation profiles in cancer treatment.

## INTRODUCTION

Protein structural integrity and stability are essential for their proper function[1, 2]. This is particularly important for proteins involved in chromatin dynamics and maintenance, as they play a critical role in regulating gene expression[3]. Within this context, the human ARID (AT-Rich Interaction Domain) family represents a physiologically significant group of proteins that participate in processes such as tissue-specific gene expression and cell growth regulation[4]. Among these, ARID1A stands out as a ubiquitous and vital component of the SWI/SNF chromatin remodeler complex[5–7], which modulates chromatin accessibility through ATP hydrolysis. Known by different nomenclatures, including B120, BAF250, BM029, and CSS2, ARID1A is crucial for organizing genomic architecture via its multidomain structure[8], influencing gene expression, DNA replication, and repair[9].

**Figure 1.**
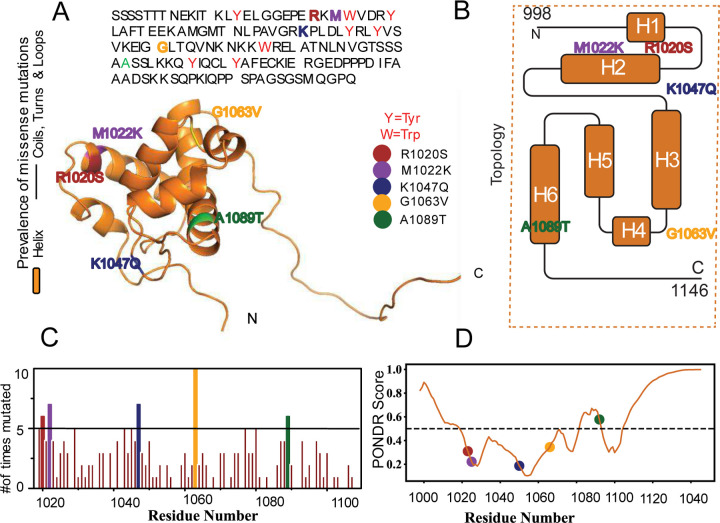
Structural and Functional Motif of the ARID Domain in ARID1A. A) Sequence and predicted three-dimensional ARID1A DNA binding domain, showing the positions of mutations and intrinsic residues as shown in color. B) Secondary structure topology of the domain, showing the position of each mutant with the same color scheme as (A). C) Frequency of every missense mutation in the ARID domain with respect to the position, using TCGA data and setting a threshold of at least five occurrences. D) Disorder prediction using the PONDR score for the ARID1A’s DNA binding domain, showing in color the position of each mutant.

ARID family members share a highly conserved DNA-binding domain, known as the ARID domain, which is approximately 100 amino acids in length[10] (Figure 1A). Structurally, this domain features a helix-turn-helix motif composed of six helices[11] (Figure 1B). The DNA-binding interface is formed by three loops near the N-terminal, along with helices H5 and H6 and their connecting loops. However, mutations within the ARID1A DNA-binding domain, common in various cancers and malignancies, pose significant challenges, including therapeutic resistance and genomic instability[12, 13]. Notably, these mutations are present in 6% of all cancers and approximately 46% of ovarian cancer cases[14, 15]. Within the ARID domain, 37% of these mutations are missense or frameshift, as documented in The Cancer Genome Atlas (TCGA)[16]. While stop-gain and frameshift mutations clearly result in loss of function[17], the pathogenic impact of missense mutations remains difficult to interpret[8]. The most frequently observed ARID missense mutations in humans are R1020S, M1022K, K1047Q, G1063V, and A1089T (Figure 1C), highlighting their potential pathogenicity. Structurally, R1020S and M1022K are located on helix H2, A1089T is found on helix H6, while the others are positioned on the loops flanking helix H3 (Figure 1B). Disruption of these regions’ structure and dynamics can impair function, potentially compromising tumor suppressor activity[18].

Using the Predictor of Natural Disordered Regions (PONDR) web server[19], we identified regions within the ARID1A protein sequence that lack a fixed tertiary structure, known as disordered regions (DR) (Figure 1D). Mutations within disordered regions could lead to dysfunction by disrupting chromatin dynamics[20]. And mutations within the structured regions could impact stability [21]. Therefore, investigating the structural consequences of these mutations is essential for understanding their physiological impact on ARID1A function.

In this study, we explored the impact of specific ARID1A mutations (Figure 1) on protein structural stability using a hybrid approach that combined computational and experimental methods. Sequence and structure-based machine learning predictions, along with replica exchange molecular dynamics simulations (rexDMD)[22], identified R1020S as the most destabilizing mutation. Experimentally, thermal and chemical denaturation assays using intrinsic fluorescence showed that R1020S significantly reduced the melting temperature $\left(T_{m}\right)$ and denaturation midpoint $\left(D_{1\text{/}2}\right)$, corroborating our computational findings. Overall, our approach provides valuable insights into how specific amino acid changes affect protein stability, suggesting these variations may be pathogenic for ARID1A.

## MATERIALS AND METHODS

### Pathogenic predictor tools

We employed several webserver tools to analyze the predicted impact of mutations on ARID1A. We used SAAFEC-SEQ [23], which applies the Pseudo-Position Specific Scoring Matrix (PsePSSM) algorithm to predict changes in protein thermodynamic stability resulting from single mutations by encoding physicochemical properties, sequence features, and evolutionary information to compute changes in free energy. INPS[24] was utilized to predict the effects of mutations on protein stability directly from the protein sequence. I-Mutant2.0Field [27] also predicted stability changes using a Support Vector Machine algorithm trained on data from the ProTherm database [25]. We also employed mCSM [26] to evaluate mutation impacts by capturing distance patterns between atoms, representing protein residue environments, and training predictive models, providing insights into protein stability and disease progression. Similarly, DDMut[27], a fast and accurate Siamese network, was used to predict changes in Gibbs Free Energy $\left(\Delta \text{G}\right)$ upon single and multiple point mutations by integrating graph-based representations with deep learning models, offering high predictive performance and scalability for understanding mutation effects and guiding rational protein engineering.

### rexDMD Simulation

In our *in-silico* investigation, all simulations were performed using all-atom discrete molecular dynamics (DMD) simulations with an implicit solvent model [22, 28] DMD is a unique molecular dynamics algorithm with significantly enhanced sampling efficiency. The interatomic interactions were modeled with discretized step functions in the Medusa force field, which has been widely discussed in previous studies[29–31]. Anderson’s thermostat was used to maintain the temperature of the simulations. The structure of wild-type ARID1A was predicted using AlphaFold 2[32]. The mutant structures were further prepared by introducing mutations at corresponding residues with the Mutagenesis tool in PyMOL [33]. We relaxed the predicted structures by performing 400 ps of DMD simulation under 300 K with a heat exchange factor of 10 to eliminate the unphysical defects. The resultant structures after relaxation were used as the starting structures for subsequent simulations.

In rexDMD simulations, multiple trajectories (replicas) run parallel under different temperatures. Temperature between replicas was exchanged every 50 ps according to the Metropolis criteria. Therefore, the simulations trapped at local energy minima could overcome the energy barrier by running at higher temperatures, thus increasing conformation sampling efficiency. In rexDMD simulations of all ARID1A mutants in this study, with a heat exchange factor of 0.1. A total of 20 trajectories with temperatures evenly distributed in the range 275–400 K lasted for ~0.65 μs, resulting in a cumulative total simulation time of ~13 μs. The minimum and maximum exchange rates between each two replicas in all simulations were 0.389 and 0.802, respectively.

The thermodynamic properties of the ARID1A variants were calculated using the weighted histogram analysis method [34] by self-consistently combining simulations under different temperatures. The potential of mean force (PMF) or effective free energy was calculated according to $PMF=-k_{B}TlnP\left(R_{PMF}\right)$ where $k_{B}$ is the Boltzmann constant, T is the simulation temperature, and $R_{PMF}$ is the multiple dimensional parameter such as radius of gyration, $R_{g}$, and $P\left(R_{PMF}\right)$ is the probability density obtained through WHAM.

### Protein expression and purification

Codon-optimized DNA sequence encoding the ARID domain of human ARID1A, and its mutants were cloned into a pET-28a (+) vector containing a His- tag, with a thrombin cleavage site(Figure S1). Mutants R1020S, M1022K, K1047Q, G1063V and A1089T mutants were generated by PCR mutagenesis using Q5 Site-Directed Mutagenesis Kit (New England Biolabs, Inc) and verified by Eton Biosciences Inc. Protein overexpression was induced in *E. coli* C41(DE3) with 0.5 mM of Isopropyl-β-D-thiogalactopyranoside (IPTG) at an optical density measured at 600 nm between 0.6–0.8. Induced cells were harvested by centrifugation and lysed under sonication and treated with DNase I from Sigma- Aldrich in 10mM PBS buffer pH 7.5 supplemented with 1mM CaCl_2_. Proteins were purified using HisPur^™^ Ni-NTA Spin Purification Kit (Thermo Scientific^™^) and eluted using the PBS buffer containing 250mM imidazole. The removal of the His tag was performed by incubating the respective protein without GuHCl with 5U of Thrombin (Cytiva) per mg of protein for two hours. This protein was further concentrated up to approximately 1–3 mg/ml and stored in −20C after verifying their size and purity through Gel Electrophoresis.

### Fluorescence measurements

The intrinsic fluorescence measurements were done in a Fluorolog by Horiba system. For all cases, 4 μM of protein concentration was excited at 275 nm and fluorescence emission was recorded between wavelengths of 290 nm to 360 nm (Excitation bandwidth 5 nm, Emission bandwidth 2.5 nm, Light source Xe lamp, response 0.5 sec).

For thermal unfolding experiments, samples were heated and constantly stirred from 293 to 363 K using a water bath at a rate of 1°C/min, where the emission spectra are recorded at intervals of 5°C. To ensure the proteins were at equilibrium during the measurements, we closely monitored and measured the kinetics between each interval of recordings of the spectra. Similarly, for the refolding experiment, each variant was cooled from 363K to 293K at the same rate for heating with the same condition applied for heating. The buffer’s spectra were recorded and subtracted as background for all measurements. Also, a magnetic stir bar is always on to ensure the samples do not aggregate.

To analyze the thermal unfolding, we estimated the apparent Tm values using Eq. 1

(1) $$fluorescence\left(x\right)=\frac{Y_{n}+Y_{d}*exp\left(\frac{\Delta H_{app}}{R}*\left(\frac{1}{T_{m}}-\frac{1}{x}\right)\right)}{1+exp\left(\frac{\Delta H_{app}}{R}*\left(\frac{1}{T_{m}}-\frac{1}{x}\right)\right)}$$

where $\Delta H_{app}$ is the apparent enthalpy change upon denaturation, $T_{\text{m}}$ is the apparent melting point, and $Y_{n}$ and $Y_{d}$ are the pre and post transition fluorescence signal.

We employed guanidine chloride (GuHCl) as a denaturant for chemical unfolding. For unfolding reactions, proteins were maintained and incubated with different GuHCl concentrations between 0–4 M at room temperature for at least 4 h to reach equilibrium. For refolding, 40 μM protein samples were incubated with 4 M of GuHCl for at least 4 h at room temperature. Next, the protein was diluted 10 times, whereas the GuHCl concentration was changed between 0–4M, incubating for at least another 4 h before taking the measurements. We measured each sample and subtracted the background using the buffer’s spectra. Also, a magnetic stir bar is always on to ensure the samples do not aggregate.

We analyzed the chemical unfolding data using Eq. 2.

(2) $$fluorescence\left(x\right)=\frac{Y_{n}+Y_{d}*exp\left(-\frac{\Delta G_{app}-m*x}{R\cdot T}\right)}{1+exp\left(-\frac{\Delta G_{app}-m*x}{R\cdot T}\right)},$$

where $\Delta G_{\text{app}}$ is the apparent unfolding free energy change, and m is the slope of denaturation transition.

### Overall Impact

We calculated the overall impact by first converting all effects into the same unit, kcal/mol, using equations (3), (4) and (5). The web server tools provided the change in folding free energy, which we denote as $\left(\Delta \Delta G_{\text{PT}}\right)$ (predictor tool). To add the effect of $\Delta T_{m}$ values from rexDMD and thermal denaturation, we used the following equations to convert it to kcal/mol:

(3) $${\text{ΔΔG}}_{\text{rexDMD}}\approx {\text{ΔH}}_{\text{m}}\left(\frac{{\text{ΔT}}_{\text{m}}}{{\text{T}}_{\text{m}}}\right)$$

(4) $$\text{and}\;\text{for}\;\text{thermal}\;\text{denaturation}\;\left(\text{TD}\right)\text{,}\;\text{ΔΔ}G_{\text{TD}}\approx \Delta H_{m}\left(\frac{\Delta T_{m}}{T_{m}}\right)$$

where $\Delta H_{m}$ is the change in enthalpy, $T_{m}$ is the melting point.

For chemical denaturation (CD), we evaluated the change in melting concentration $\left(\Delta D_{1\text{/}2}\right)$ and used

(5) $$\text{ΔΔ}G_{CD}\approx m\times \Delta D_{1\text{/2}}$$

where m is the slope of denaturation curve and $D_{1\text{/}2}$ is the denaturant concentration at which half of native signal is lost. Finally, we summed all the ΔΔG values and normalized the result by dividing by the reference value (the largest individual change).


(6) $$\text{Overall}\;\text{Impact}=\frac{\text{|ΔΔ}G_{PT}\text{|}+\text{|ΔΔ}G_{rexDMD}\text{|}+\text{|ΔΔ}G_{TD}\text{|}+\text{|ΔΔ}G_{CD}\text{|}}{\text{scaling}\;\text{factor}}$$

### Circular dichroism experiments

Measurements were done in a J-1500 spectropolarimeter instrument using 10 μM of protein concentration, recording the spectra from 200 to 260 nm at different 2,2,2-trifluoroethanol (TFE) concentrations (0–50% v/v), incubating at room temperature. The scanning rate was 50 nm/min.

## RESULTS

### R1020S is the most destabilizing mutation by pathogenicity prediction tools

**Figure 2.**
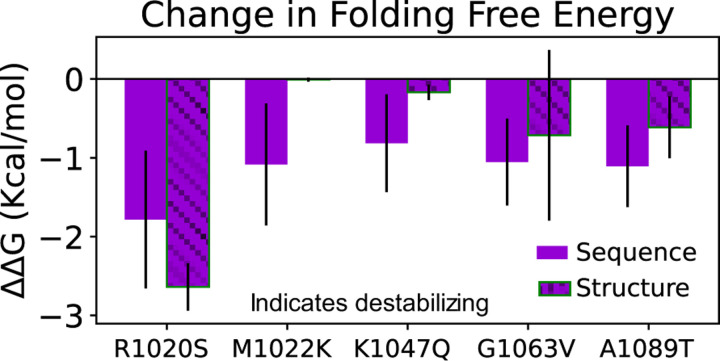
Stability changes in ARID1A predicted by both sequence-based and structure-based models with respect to the selected mutations. The position and type of mutation are shown by the x-axis along with the unhatched from sequence-based and hatched from structure-based predictors. The evaluation of stability $\left(\Delta \Delta {\text{G}}_{\text{folding}}\right)$ was made by calculating $\Delta {\text{G}}_{\text{WT}}\text{-}\Delta {\text{G}}_{\text{MUT}}$. Most of the mutations of interest show a negative $\Delta \Delta {\text{G}}_{\text{folding}}$ value, suggesting that the mutations are destabilizing.

We evaluated the effects of five missense mutations (R1020S, M1022K, K1047Q, G1063V, and A1089T) on ARID1A’s stability by employing sequence-based predictors (SAAFEC-SEQ, INPS, and I-Mutant2.0) and structure-based predictors (mCSM and DDMut) (see Materials and Methods). We compared the predicted stability and the change in folding free energy $\left(\Delta \Delta G\right)$ of these mutations against the wild type $\left(\Delta \Delta \text{G=}\Delta \text{GWT-}\Delta \text{GMUT}\right)$,interpreting negative values as destabilizing and positive values as stabilizing. Most mutations predominantly resulted in destabilization, although a few exhibited minor stabilizing effects (Figure 2 and Figure S2). Remarkably, the R1020S mutation had the most significant destabilizing impact across both sequence-based and structure-based predictors. However, the heterogeneity in predicted effects emphasizes the complexity of the mutational landscape and the challenges of relying solely on web server tools for accurate predictions.

### Discrete Molecular Dynamics Simulations showed the R1020S as the most destabilizing mutant.

**Figure 3.**
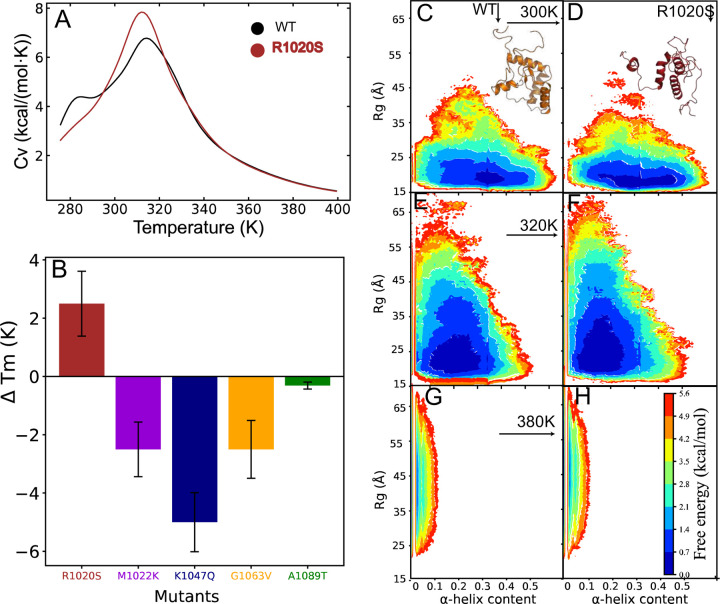
rexDMD simulations for wild-type and mutants. A) variation of $C_{v}$ and temperature for the WT and R1020S, B) Change in melting temperature $\left(\Delta T_{m}\right)$ for all mutants obtained from $C_{v}$ calculation, C-H) 2D-PMF plot of WT (C, E and G) and R1020S (D, F and H) at different temperatures (top panel 300 K, middle panel 320 K and bottom panel 380K) of $R_{g}$ versus the α-helical contents. The structures of in C and D are the native representation (basin at ~0.32 α-helical content and ~17 Å of $R_{g}$).

To illustrate the impact of mutations on protein stability and dynamics at the molecular level, we conducted replica-exchange discrete molecular dynamics (rexDMD) simulations of both wild-type (WT) and mutant proteins. We ran 20 replicas at consecutive temperatures (300–380 K) over a cumulative time. To assess the changes in structural dynamics and impact introduced by the mutations induced by heating, we calculated the specific heat $\left(C_{v}\right)$ and the average radius of gyration $\left(R_{g}\right)$ within the simulated temperature range for all proteins (WT and mutants). The $C_{v}$ curves revealed that, except for the R1020S mutant, all amino acid substitutions were stabilizing compared to the WT protein (Figure 3A, Figure S3A). Next, we determined the temperature dependence of $R_{g}$ across the specified temperature range (see Materials and Methods). The unfolding process exhibited cooperativity in all cases, indicated by the sigmoidal curves (Figure S3B). We estimated the melting temperature as the temperature corresponding to the peak of $C_{v}$ and calculated the difference in melting temperatures $\left(\Delta T_{m}=T_{m,WT}-T_{m,MUTANT}\right)$ for all proteins. Only the R1020S mutation showed a destabilizing effect (Figure 3B). Given the consistent destabilizing effect of the R1020S mutant, we aimed to use a high-resolution approach to further understand its impact on stability.

We further examined the free energy landscape by analyzing the potential mean force in relation to α-helix content and radius of gyration $\left(R_{g}\right)$ for the wild type (WT) and the R1020S mutant (Figure 3, C–H, Figure S4). At 300 K, the WT protein exhibited an energy minimum with an α-helix content of approximately 0.32 and an $R_{g}$ of 17 Å. In contrast, the R1020S mutant showed a much broader distribution in α-helix content, indicating greater structural heterogeneity. As the temperature increased, both variants demonstrated a decrease in α-helix content and an increase in $R_{g}$, reflecting increased structural flexibility due to partial unfolding. However, thermal denaturation occurred differently among the mutants at 320 K. Notably, the R1020S mutant exhibited a wider range of $R_{g}$, consistent with its reduced thermal stability. At 380 K, we observed a complete loss of α-helices and a significant increase in $R_{g}$, indicating the full unfolding of the WT and mutant ARID1A. Overall, our simulations confirmed that the R1020S mutation was the most destabilizing, significantly increasing structural heterogeneity and reducing the thermal stability of ARID1A.

### Thermal stability showed irreversible process with R1020S impactful mutation

Bioinformatic approaches and molecular dynamics simulations suggest that the R1020S destabilizes the protein the most compared to the other mutations, likely due to the increased structural heterogeneity. To validate these predictions, we experimentally assessed the protein’s stability through thermal denaturation using intrinsic fluorescence measurement. We monitored changes in fluorescence across a temperature range of 298–360 K.

**Figure 4.**
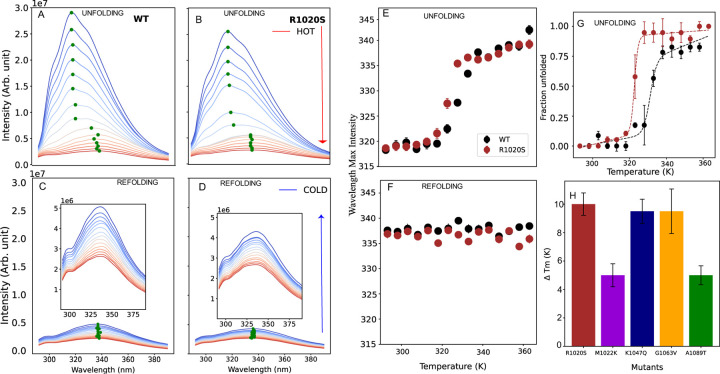
Thermal denaturation experiment with wild-type ARID1A and mutants: A) and B) show unfolding, whereas C) and D) show refolding spectra plots obtained for WT and R1020S (the inset fig to zoom in view with different scale for clarity). E) and F) show the wavelength shift at max intensity for WT and R1020S as a function of temperature for unfolding and refolding, respectively. G) normalized unfolding curve to estimate apparent melting point for wild-type and R1020S mutant, using a two-state model (Material and Methods). H) Difference in melting temperature $\left({\text{ΔT}}_{\text{m}}={\text{T}}_{\text{m,WT}}\text{-}\;{\text{T}}_{\text{m,MUT}}\right)$ obtained from all mutants.

We began with unfolding experiments, which revealed a red shift in the emission wavelength at maximum intensity and a decrease in fluorescence intensity in all cases (Figure 4A and B, Figure S5). Next, we assessed the reversibility of the process by conducting refolding experiments over the same temperature range, starting with protein previously incubated at 360–298 K. During subsequent refolding, the variants exhibited a minimal blue shift in wavelength at maximum intensity, indicating irreversibility, and only partial recovery of fluorescence intensity (Figure 4C and D, Figure S5). Notably, the R1020S mutant showed less fluorescence recovery compared to WT, and the native maximum wavelength was not restored in any case.

Given the irreversible nature of thermal unfolding (Figure 4E and F, Figure S6A and B), we analyzed the fluorescence data by fitting it to a two-state unfolding model to determine the apparent melting temperature $\left(T_{m,app}\right)$ (Materials and Methods). We then compared these values to the WT $\left(\Delta T_{m}=T_{m,WT}-T_{m,MUT}\right)$ to assess stability differences (Figure 4G, Figure S6C). The extracted $T_{m,app}$ varied among the mutants (Figure S6D), with WT displaying the highest thermal stability at $T_{m,app}$ of 333±1 K, indicating that all mutations are destabilizing. The R1020S, K1034Q, and G1063V mutants exhibited the lowest stabilities, with average $\Delta T_{m}$ values decreasing by 10 K (Figure 4H). Overall, these thermal unfolding results align with bioinformatics predictions and rexDMD findings, confirming the significant destabilizing impact of the R1020S mutation on the protein’s stability.

### Chemical stability showed reversibility and R1020S the most impactful mutation

Our thermal denaturation experiments showed that the protein underwent irreversible unfolding, yet we could obtain apparent melting values that supported the bioinformatic predictions. To determine whether the unfolding process is reversible under chemical denaturation, we conducted experiments using 0 to 4 M GuHCl (Materials and Methods) to induce protein unfolding and refolding, while monitoring the intrinsic fluorescence.

**Figure 5.**
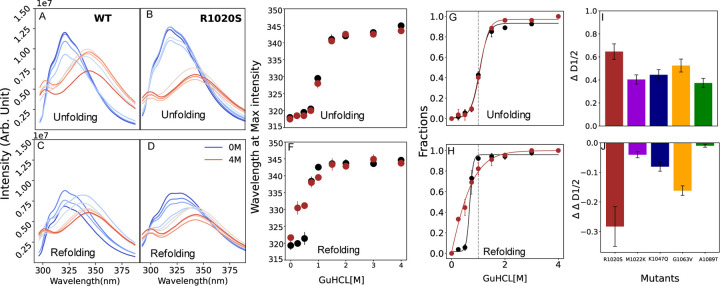
Chemical denaturation experiment with wild-type ARID1A and mutants. A) and B) show unfolding, whereas C) and D) show refolding spectra plots obtained for WT and R1020S. E) and F) show the wavelength shift at max intensity for WT and R1020S as a function of GuHCl for unfolding and refolding, respectively. G) and H) normalized unfolding and refolding curve to estimate apparent $D_{1\text{/}2}$ for wild-type and R1020S mutant, using a two-state model (Material and Methods). I) and J) Difference in $D_{1\text{/2}}\left(\Delta D_{1\text{/2}}=D_{1\text{/2}unfolding}-D_{1\text{/2}refolding}\right)$ and Difference in $\Delta D_{1\text{/2}}\left(\text{ΔΔ}D_{1\text{/2}}=\Delta D_{1\text{/2},WT}-\Delta D_{1\text{/2},mutant}\right)$ obtained from all mutants.

During unfolding, we initially observed a slight increase to no effect in the fluorescence intensity at low GuHCl concentrations, followed by a decrease as unfolding progressed. For all mutants, a red shift in the emission wavelength at maximum intensity was recorded with increasing GuHCl concentration (Figure 5A and B, Figure S7). To assess the reversibility of this process, we conducted refolding experiments, observing a blue shift in the emission wavelength back to its original maximum (Figure 5C and D, Figure S7), with over 80% of fluorescence intensity being restored. However, this partial recovery may be attributed to the extended exposure of the samples at room temperature, which nearly doubled the equilibration time for refolding (unfolding followed by refolding). We plotted the wavelength shift at maximum intensity against GuHCl concentration (Figure 5E and F, Figure S8A and B) and normalized the data, fitting it to a two-state model (Materials and Methods) for both unfolding and refolding, clearly demonstrating reversibility (Figure 5G and H, Figure S8C and D).

Interestingly, the unfolding and refolding pathways differed, as indicated by the $D_{1\text{/}2}$ values, which represent the GuHCl concentrations at which 50% of the protein is unfolded or refolded. We calculated the change in $\Delta D_{1\text{/2}}=D_{1\text{/2}unfolding}-D_{\text{1/2}refolding}$ (Figure 5I) for all mutants, with WT showing a $\Delta D_{1\text{/}2}$ of 0.365 (not shown in the figure). The R1020S mutant exhibited the largest difference in its $\Delta D_{1\text{/}2}$ value, indicating high instability in response to chemical denaturation. We then calculated $\text{ΔΔ}D_{1\text{/2}}=\Delta D_{1\text{/2},WT}-\Delta D_{1\text{/2},mutant}$ (Figure 5J), revealing that R1020S had the largest $\Delta \Delta D_{1\text{/}2}$ difference, consistent with results from other methods, highlighting it as the most affected by chemical denaturation. Surprisingly, the A1089T mutant had the smallest $\Delta D_{1\text{/}2}$ and $\Delta \Delta D_{1\text{/}2}$ values, suggesting a different response to chemical denaturation.

## DISCUSSION

In this article, we evaluated the most prevalent ARID1A mutations to assess their impact on the protein’s stability. By combining computational and experimental approaches, we found that the R1020S mutation causes the greatest destabilization. In contrast, the M1022K mutation exhibits the highest stability, as further supported by secondary structure content and CD spectra (Figure S3C and S9).

**Figure 6.**
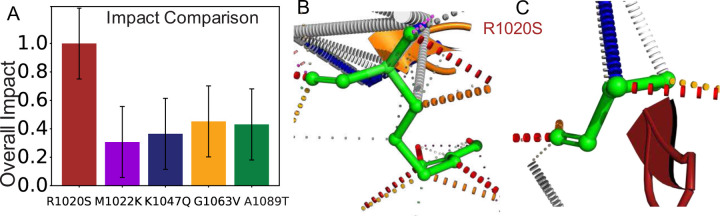
The overall impact showed R1020S the most impacted. A) The overall impact as weighted average of the computational and experimental outcomes. B)WT interacting sites at R1020S(green) show a greater number of interaction sites, C) The interacting sites in the mutant at R1020S((green) )show fewer interacting sites.

To evaluate the overall impact of the selected ARID1A mutations on protein stability and potential for pathogenicity, we computed a weighted average of all determined parameters (see Materials and Methods). This categorization agrees with the Pearson coefficient of 0.66 with the predictions made by the Alpha Missense (Figures 6A, S12), a machine learning tool developed by Deep Learning, which assesses the pathogenicity of 216 million possible single amino acid changes across human proteins. AlphaMissense provides highly accurate classifications, supporting diagnostics and research on complex traits [35–37]. Our classification approach enhances the prediction of structural and functional impairments, which is essential for understanding disease mechanisms associated with protein structural instability. These insights can be translated into targeted therapeutic strategies, refining treatments tailored to individual genetic profiles, thereby advancing personalized medicine and promoting progress in drug discovery during clinical trials [38, 39].

Given that most mutations occur in regions with high propensity for α-helical content, it’s noteworthy that only the R1020S mutant significantly impacts stability, suggesting that inter amino acid interactions may drive this effect. We used the structures predicted by DDMut[27], which identifies detailed interaction sites within the three-dimensional context, to get insights into the number of interactions. These structural models (Figure 6B and 6C) revealed that the R1020S mutation disrupts salt bridges with negatively charged residues within the same helix (H2) and helix H3, potentially altering bonding and leading to the observed instability. In contrast, the M1022K mutation introduces a positively charged residue that can form new salt bridges, enhancing stability and contributing to a more rigid structure. Although other mutations affect the α-helical content, they maintain their stability, suggesting that factors such as structural conservation of the DNA binding might be responsible for their pathogenicity. In a further study, we are exploring the direct impact on the binding free energy due to these missense mutations.

While predictive tools like web servers provide valuable initial insights, they often fall short in capturing the full biophysical and biochemical complexities of proteins, particularly in varied environmental conditions. For example, our experiments revealed significant differences between thermal and chemical denaturation, underscoring the biological relevance of these distinctions.

This is critical because web servers typically assume reversible folding, a simplification that may not accurately represent the behavior of mutations susceptible to irreversible unfolding under thermal or chemical stress. Relying solely on these tools can lead to incomplete or misleading conclusions. Thus, integrating complementary experimental and computational approaches is essential for a thorough understanding of protein stability and the biological impacts of mutations and paves the way for identifying and categorizing which mutations are most pathogenic, potentially guiding the development of targeted therapies tailored to specific mutation profiles in cancer treatment.

## Supplementary Material

Supplement 1

## Data Availability

Source files for rexDMD simulations and the intrinsic fluorescence spectra generated are available with the accession code doi:10.5281/zenodo.13712720.
