# Supplementary material for: Impact of Frequent ARID1A Mutations on Protein Stability: Insights into Cancer Pathogenesis
Source: Res Sq. 2024 Dec 19:rs.3.rs-5225582. Preprint. [Version 1] doi: 10.21203/rs.3.rs-5225582/v1 (PMC11702796; doi:10.21203/rs.3.rs-5225582/v1)
Supplement: Supplement 1 [file NIHPPRS5225582v1-supplement-1.pdf]

Supplementary Files

This is a list of supplementary files associated with this preprint. Click to download.

- SIv1HS.docx
